# Anti-tumor activity of selective inhibitors of XPO1/CRM1-mediated nuclear export in diffuse malignant peritoneal mesothelioma: the role of survivin

**DOI:** 10.18632/oncotarget.3761

**Published:** 2015-04-18

**Authors:** Michelandrea De Cesare, Denis Cominetti, Valentina Doldi, Alessia Lopergolo, Marcello Deraco, Paolo Gandellini, Sharon Friedlander, Yosef Landesman, Michael G. Kauffman, Sharon Shacham, Marzia Pennati, Nadia Zaffaroni

**Affiliations:** ^1^ Molecular Pharmacology Unit, Department of Experimental Oncology and Molecular Medicine, Fondazione IRCCS Istituto Nazionale dei Tumori, Milano, Italy; ^2^ Peritoneal Surface Malignancy Program, Department of Surgery, Fondazione IRCCS Istituto Nazionale dei Tumori, Milano, Italy; ^3^ Karyopharm Therapeutics Inc., Newton, MA, USA

**Keywords:** diffuse malignant peritoneal mesothelioma, SINE, survivin, XPO1/CRM1

## Abstract

Survivin, which is highly expressed and promotes cell survival in diffuse malignant peritoneal mesothelioma (DMPM), exclusively relies on exportin 1 (XPO1/CRM1) to be shuttled into the cytoplasm and perform its anti-apoptotic function. Here, we explored the efficacy of Selective Inhibitors of Nuclear Export (SINE), KPT-251, KPT-276 and the orally available, clinical stage KPT-330 (selinexor), in DMPM preclinical models. Exposure to SINE induced dose-dependent inhibition of cell growth, cell cycle arrest at G1-phase and caspase-dependent apoptosis, which were consequent to a decrease of XPO1/CRM1 protein levels and the concomitant nuclear accumulation of its cargo proteins p53 and CDKN1a. Cell exposure to SINE led to a time-dependent reduction of cytoplasmic survivin levels. In addition, after an initial accumulation, the nuclear protein abundance progressively decreased, as a consequence of an enhanced ubiquitination and proteasome-dependent degradation. SINE and the survivin inhibitor YM155 synergistically cooperated in reducing DMPM cell proliferation. Most importantly, orally administered SINE caused a significant anti-tumor effect in subcutaneous and orthotopic DMPM xenografts without appreciable toxicity. Overall, we have demonstrated a marked efficacy of SINE in DMPM preclinical models that may relay on the interference with survivin intracellular distribution and function. Our study suggests SINE-mediated XPO1/CRM1 inhibition as a novel therapeutic option for DMPM.

## INTRODUCTION

Diffuse malignant peritoneal mesothelioma (DMPM) is an uncommon and locally aggressive tumor that develops from mesothelial cells lining the peritoneal cavity, and accounts for approximately 25–30% of all mesotheliomas [1, 2]. The prognosis of DMPM is poor and treatment of DMPM patients by palliative surgery, systemic/intraperitoneal chemotherapy and abdominal irradiation showed to be ineffective with a median survival of about one year [2–4]. The advent of a loco-regional strategy that combines aggressive cytoreductive surgery (CRS) with hyperthermic intraperitoneal chemotherapy (HIPEC) [3,4] significantly improved median survival up to 40–92 months in selected series of patients, although approximately 40–60% of patients still experience recurrence [3–5]. For these patients, and for those who are not eligible to CRS+HIPEC, the prognosis remains severe due to the lack of effective alternative treatment options, highlighting the need to develop new therapeutic strategies.

Previous work from our lab suggests that dysregulation of the apoptotic pathway may play a role in DMPM resistance to chemotherapy and that survivin and other Inhibitors of Apoptosis Protein (IAP) family members may represent new therapeutic targets [6]. Indeed, we found that RNAi-mediated survivin knockdown in DMPM cells enhanced both spontaneous and cytotoxic drug-induced apoptosis [6], thus supporting the notion that agents targeting survivin may provide new treatment approaches for this disease. Survivin is a structurally unique member of the IAP family and it is involved both in the control of cell division and inhibition of the apoptotic machinery [7]. Notably, sub-cellular compartmentalization of survivin plays an essential role in determining its bifunctional role [8]. Nuclear localization of survivin is mainly involved in spindle monitoring at mitosis, whereas cytoplasmic/mitochondrial survivin counteracts pro-apoptotic signals by preventing caspase-9 and caspase-3 activation [8]. While the low molecular weight of survivin allows its passive diffusion from the cytoplasm to the nucleus, its export from the nucleus back to the cytoplasm requires an interaction between the exportin-1/chromosome maintenance protein 1 (XPO1/CRM1) and the specific leucine-rich nuclear export signals (NES) within survivin. This interaction is accomplished via the RanGTP/GDP axis [8, 9]. XPO1/CRM1 is a key member of the importin β superfamily of nuclear transport receptors that are involved in the nucleo-cytoplasmic active transport of over 200 proteins, including transcription factors, tumor suppressors, cell-cycle regulators and proteins involved in programmed cell death [10, 11].

Recently, a novel class of oral bioavailable small-molecule Selective Inhibitors of Nuclear Export (SINE) has been developed. These inhibitors bind specifically to the NES-binding groove of XPO1/CRM1 and prevent the interaction with its cargo proteins [12–14]. These compounds have demonstrated anti-tumor activity in a variety of experimental models of solid and hematologic malignancies both *in vitro* and *in vivo* [12, 13, 15–29]. Among those, selinexor (KPT-330) is the most advanced SINE with >500 hematologic and solid cancer patients treated to date in a number of Phase I/II clinical trials. (http://www.clinicaltrials.gov).

In the present study we investigated the therapeutic potential of three SINE, namely KPT-251, KPT-276 and selinexor, in patient-derived DMPM experimental models. Our results show that XPO1/CRM1 inhibition significantly impairs DMPM cells growth *in vitro* and *in vivo*, by inducing a marked apoptotic response. Furthermore, we provide evidence that SINE exert their pro-apoptotic effect by controlling the sub-cellular localization of survivin and subsequently modulating its expression through an ubiquitin/proteasome-dependent mechanism.

## RESULTS

### SINE impair DMPM cell growth

**Figure 1:**
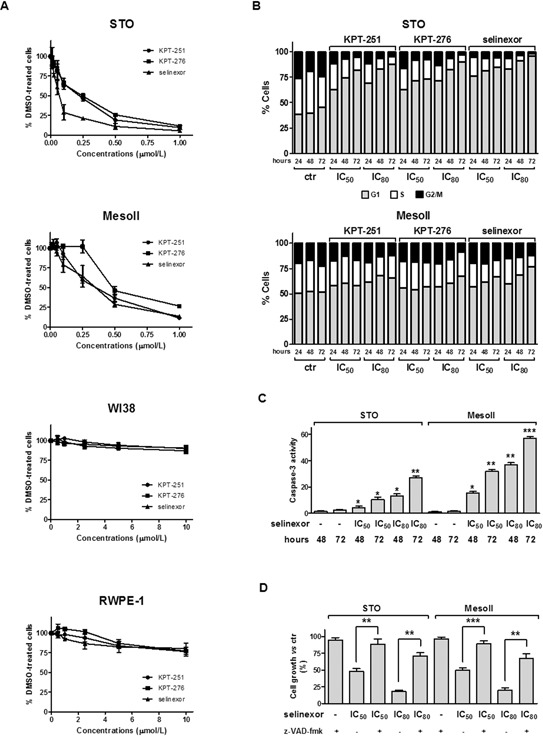
SINE impair cell growth, promote cell cycle arrest, and induce apoptosis in DMPM cells **A.** Cytotoxic activity of SINE in DMPM (STO and MesoII) and human normal (WI38 and RWPE-1) cell lines. Cells were cultured for 72 hours in the presence of increasing concentrations of SINE, and the cytotoxic activity was assessed by MTS assay. Data are expressed as mean values ±SD of at least three independent experiments. **B.** Flow-cytometric analysis of DMPM cells stained with propidium iodide at different intervals (24, 48, and 72 hours) after treatment with 0.01% DMSO (ctr) or SINE (IC_50_ and IC_80_, which were determined graphically from the dose-response curves obtained after a 72-hour exposure of cells to SINE in the MTS assay). Data are reported as the percentage of cells in G1, S and G2/M phases and represent the mean values of three independent experiments; SDs were always within 5%. **C.** Assessment of caspase-3 catalytic activity at 48 and 72 hours after treatment with 0.01% DMSO (ctr) or selinexor (IC_50_ and IC_80_, which were determined graphically from the dose-response curves obtained after a 72-hour exposure of cells to the drug in the MTS assay). Data are expressed as relative fluorescence units and represent the mean values ±SD of at least three independent experiments. **D.** Cytotoxic effect of selinexor in DMPM cells after pre-incubation with z-VAD-fmk. Cells were cultured for 72 hours with selinexor (IC_50_ and IC_80_, which were determined graphically from the dose-response curves obtained after a 72-hour exposure of cells to SINE) in the presence/absence of z-VAD-fmk, and the cytotoxic activity was assessed by MTS assay. Data are expressed as percentage values of growth in treated cells compared with cells exposed to 0.01% DMSO (ctr), and represent mean values ±SD of at least three independent experiments. ***P < 0.001, **P < 0.01, *P < 0.05.

The effect of SINE on cell growth of two human DMPM cell lines (STO and MesoII, expressing wild-type and mutant *TP53*, respectively) (Supplementary Table 1), was assessed by MTS assay following exposure to increasing concentrations of KPT-251, KPT-276 or selinexor. A dose- and time-dependent inhibition of cell growth was consistently observed in both cell lines after treatment with the different compounds (Figure 1A and Supplementary Figure S1A, B). However, while STO cells showed a higher sensitivity to selinexor compared to KPT-251 and KPT-276, with IC_50_ values of 0.07 ± 0.01, 0.23 ± 0.05 and 0.24 ± 0.02 μmol/L respectively, MesoII cells showed a comparable sensitivity to all the compounds with IC_50_ values of 0.35 ± 0.09, 0.36 ± 0.04 and 0.47 ± 0.04 μmol/L, respectively. In addition, at concentrations up to 10 μmol/L, SINE did not alter the growth of both normal human lung fibroblast (WI38) and adult human prostate (RWPE-1) cell lines (Figure 1A).

### SINE promote cell cycle arrest and induce a caspase-dependent apoptotic cell death in DMPM cells

Since XPO1/CRM1 mediates nuclear export of several cell cycle regulatory proteins, including p53, cyclin B1, cyclin D1, cyclin-dependent kinase inhibitor 1a (CDKN1a) and cyclin-dependent kinase inhibitor 1b (CDKN1b) [9, 11], we set to determine the effect of SINE on cell cycle progression. DMPM cells were exposed to KPT-251, KPT-276 or selinexor (at predetermined IC_50_ and IC_80_ of each cell line), and stained with propidium iodide at 24, 48 and 72 hours-post treatment. Flow cytometry profiles of nuclear DNA content revealed that 24-hour treatment of STO cells with SINE was sufficient to induce an accumulation of cells in G1 phase and a reduction in the percentage of cells in S and G2/M compartments (Figure 1B). G1 phase accumulation markedly increased at 48 hours and reached a maximum 72 hours-post exposure to the highest doses of SINE (87.6 ± 3.7%, 90.4 ± 1.8% and 96.1 ± 3.3% for KPT-251, KPT-276, and selinexor, respectively) (Figure 1B). Although to a lesser extent compared to STO cells, an increase in the percentage of cells in G1 phase was appreciable following 72-hour exposure to the highest selinexor concentration in MesoII cells (Figure 1B).

To verify whether SINE-induced tumor cell growth inhibition was also dependent on the induction of an apoptotic cell death, we analyzed the presence of Annexin V^+^ cells 48 and 72 hours-post drug exposure by flow cytometry. While the apoptotic cell fraction was <10% in control cells at both time points, a marked dose- and time-dependent increase in the percentage of Annexin V^+^ cells was observed in the treated STO and MesoII cells (Table 1 and Supplementary Figure S2). In addition, a significant dose- and time-dependent increase in caspase-3 catalytic activity, as determined *in vitro* by the hydrolysis of the specific fluorogenic substrate, was found after treatment with each compound (Figure 1C and Supplementary Figure S3). Specifically, in STO cells exposed for 72 hours to KPT-251, KPT-276 and selinexor (IC_80_), the catalytic activity of caspase-3 was 7-, 6- and 11-fold higher, respectively, than that observed in control samples (Figure 1C and Supplementary Figure S3A). Similarly, a 21-, 23- and 33-fold increase in caspase-3 catalytic activity was also observed in MesoII cells treated with KPT-251, KPT-276 and selinexor, respectively (Figure 1C and Supplementary Figure S3A). Notably, the inhibitory effect of SINE on cell growth was almost completely reverted when DMPM cells were pretreated with the pan-caspase inhibitor z-Val-Ala-Asp-fluoromethylketone (z-VAD-fmk; Figure 1D and Supplementary Figure S3B) -which by itself failed to impair cell growth (Figure 1D)-, providing evidence that SINE induce a caspase-dependent apoptotic cell death in DMPM cells.

**Table 1:** Induction of apoptosis in DMPM cells treated with KPT-251, KPT-276 and selinexor

Treatment		STO		MesoII	
		48 hours	72 hours	48 hours	72 hours
ctr		5.1 ± 0.7	3.3 ± 0.5	9.0 ± 2.1	3.2 ± 1.1
KPT-251	IC_50_	8.6 ± 1.7^*^	18.6 ± 2.5^**^	14.5 ± 1.6^**^	22.1 ± 0.3^***^
	IC_80_	14.5 ± 1.2^**^	22.6 ± 0.9^***^	22.2 ± 1.5^***^	30.5 ± 0.8^****^
KPT-276	IC_50_	7.9 ± 0.4^*^	17.0 ± 2.0^**^	19.6 ± 2.2^***^	26.4 ± 4.5^***^
	IC_80_	13.0 ± 1.2^**^	25.6 ± 1.0^***^	27.1 ± 1.1^***^	45.5 ± 2.1^****^
selinexor	IC_50_	11.3 ± 2.9^*^	21.5 ± 2.8^***^	22.3 ± 3.2^***^	37.5 ± 1.1^****^
	IC_80_	12.7 ± 1.3^*^	28.9 ± 1.9^***^	33.0 ± 2.0^****^	61.7 ± 1.7^****^

Induction of apoptosis was evaluated by flow cytometry as the presence of Annexin V positive cells (i.e., Annexin V+/PI- plus Annexin V+/PI+ cells) after 48 and 72 hours of treatment with 0.01% DMSO (ctr) or SINE (IC_50_ and IC_80_, which were determined graphically from the dose-response curves obtained after a 72-hour exposure of cells to the different compounds in the MTS assay). Data represent mean values ±SD of at least three independent experiments.

**** *P* < 0.0001,

*** *P* < 0.001,

** *P* < 0.01,

* *P* < 0.05

### SINE modulate nuclear levels of XPO1/CRM1 and its cargo proteins

**Figure 2:**
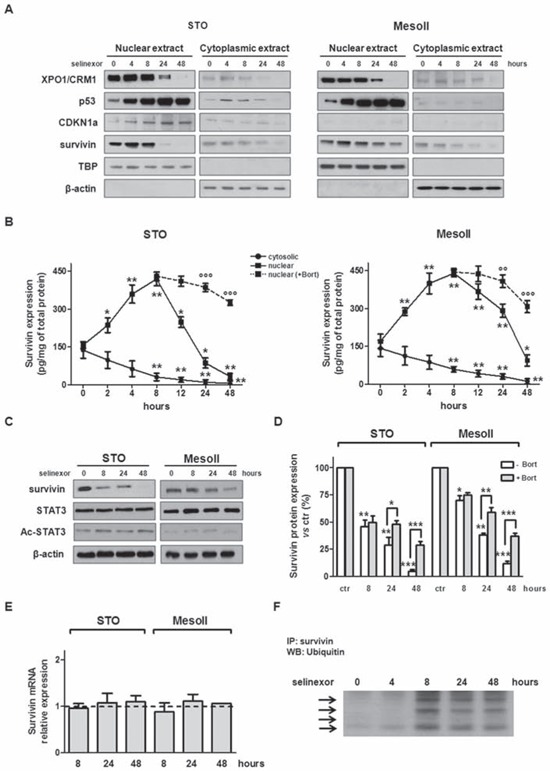
SINE inhibit XPO1/CRM1 functions, interfere with survivin subcellular distribution and promote its proteosome-dependent degradation **A.** Representative western immunoblotting showing nuclear and cytosolic fractions of XPO1/CRM1, p53, CDKN1a and survivin in DMPM cells exposed to selinexor (IC_50_). β-actin and TBP were used to confirm equal protein loading on the gel and to show the relative purity of the nuclear fractions. **B.** Quantification of nuclear and cytosolic survivin protein levels by ELISA assay in DMPM cells exposed to selinexor (IC_50_) alone or in the presence of subtoxic concentrations of Bortezomib. Data are reported as amount (pg) of survivin normalized to total (mg) protein, and represent the mean values ±SD of at least three independent experiments. **C.** Representative western immunoblotting showing the expression of survivin, STAT3 and Ac-STAT3 in DMPM cells exposed to selinexor (IC_50_). β-actin was used to confirm equal protein loading on the gel. **D.** Quantification of survivin protein levels by ELISA assay in DMPM cells exposed to selinexor alone (IC_50_) or in the presence of subtoxic concentrations of Bortezomib (1 nmol/L). Data are reported as the percentage of survivin expression in selinexor-treated cells compared with cells exposed to 0.01% DMSO (ctr), and represent the mean values ±SD of at least three independent experiments. **E.** Quantification of survivin mRNA expression levels by qRT-PCR in DMPM cells exposed to selinexor (IC_50_). Data are reported as log10-transformed relative quantity (RQ) in selinexor-treated cells with respect to cells exposed to 0.01% DMSO (ctr), and represent the mean values ±SD of at least three independent experiments. Dashed line: relative survivin mRNA expression level in the ctr. **F.** Representative IP experiment showing increased ubiquitination of nuclear survivin in STO cells exposed to selinexor (IC_50_) ***P < 0.001, **P < 0.01, *P < 0.05, *vs* ctr; °°°*P* < 0.001, °°*P* < 0.01, °*P* < 0.05, cells exposed to selinexor *vs* cells exposed to selinexor+Bortezomib.

To better understand the mechanism underlying SINE cytotoxic effect, we determined the levels of expression of XPO1/CRM1 and its cargo proteins p53 and CDKN1a before and after treatment. Consistently with previous works in different tumor type models [13, 17, 19, 21–23, 25], immunoblotting analysis revealed that nuclear XPO1/CRM1 expression progressively decreased after SINE treatment (Figure 2A and Supplementary Figure S4). In addition, the compounds induced nuclear accumulation of p53 as early as 4 hours-post treatment initiation in both cell lines, whereas CDKN1a nuclear accumulation was observed only in STO cells (Figure 2A and Supplementary Figure S4).

### SINE interfere with the subcellular localization of survivin and induce its down-regulation through the ubiquitin/proteosome pathway

Survivin is a key anti-apoptotic protein and a cargo of XPO1/CRM1 [7–9]. Previous work has shown that its subcellular localization determines its function [8, 10]. Therefore, we first assessed the effect of SINE on the subcellular compartmentalization of survivin by Western blot and ELISA. Interestingly, SINE treatment (at IC_50_) induced nuclear accumulation of survivin concomitant with a time-dependent cytoplasmic reduction (Figure 2A, 2B and Supplementary Figure S5). Survivin nuclear accumulation was observed as early as 2 hours-post exposure to each compound and it reached a maximum 8 hours-post treatment initiation. Strikingly, starting from 12 hours-post treatment initiation, a progressive decrease in nuclear survivin protein abundance was observed (Figure 2A, 2B and Supplementary Figures S5 and S6), resulting in a significant and time-dependent reduction of total protein amount (Figure 2C, 2D).

It has been recently shown in triple-negative breast cancer (TNBC) cells that inhibition of XPO1/CRM1 by selinexor represses *survivin* transcription by inhibiting STAT3 acetylation [22]. We therefore assessed STAT3 protein expression and acetylation in DMPM cells following selinexor treatment by Western blot (Figure 2C). However, no measurable effects on protein levels and acetylation status were observed. Our data suggest that the decrease of survivin protein abundance in DMPM cells is not related to post-translational modifications of its well-known transcriptional activator. Such a hypothesis is also corroborated by the evidence that exposure of DMPM cells to selinexor did not affect survivin mRNA expression (Figure 2E).

Since it has been reported that the forced retention of survivin in the nucleus promotes its clearance by the ubiquitin-proteasome proteolytic pathway [30], we checked whether selinexor-mediated XPO1/CRM1 inhibition might lead to the ubiquitination of survivin nuclear fraction. Western blot experiments indicated that exposure of DMPM cells to selinexor resulted in multiple ubiquitination of survivin, which increased its molecular weight up to 100 kDa (Figure 2F). These results suggest that in DMPM cells the reduction of survivin nuclear fraction by selinexor is ascribable at least in part to its proteasome-dependent degradation. Indeed, exposure of STO cells to the proteosome inhibitor Bortezomib partially restored nuclear survivin levels in selinexor treated cells (Figure 2B, 2D and Supplementary Figure S6).

### SINE synergistically cooperate with YM155 to inhibit DMPM cell proliferation

**Figure 3:**
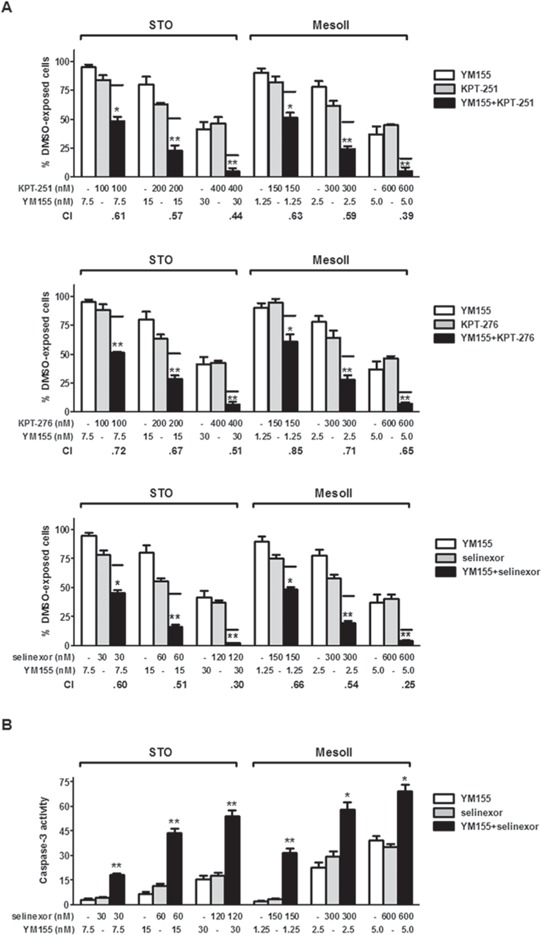
Synergistic cytotoxic effect of SINE/YM155 combinations **A.** Cytotoxic effect of KPT-251 (up), KPT-276 (middle) and selinexor (down) in combination with YM155. DMPM cells were exposed to SINE and YM155 for 72 hours, and the cytotoxic effect was assessed by MTS assay. Black lines represent the expected additive effect of the combination, calculated as the product of the effects of the individual drugs, according to the method of Kern *et al* [46]. Data are expressed as percentage values of growth in treated cells compared to cells exposed to 0.01% DMSO (ctr), and represent mean values ±SD of at least three independent experiments. CI was calculated according to Chou and Talalay [45]. **B.** Assessment of caspase-3 catalytic activity at 72 hours after treatment with selinexor and YM155, alone and in combination. Data are expressed as relative fluorescence units and represent the mean values ±SD of at least three independent experiments. **P < 0.001, *P < 0.01, *vs* single treatments.

The combined effects of SINE and the survivin inhibitor YM155 -found to induce a time-dependent survivin decrease at both mRNA and protein level (Supplementary Figure S7)- were investigated in DMPM cells. In combination experiments, cells were simultaneously exposed to increasing concentrations of SINE and YM155for short (72 hours) and long (10 days) time periods, and the cytotoxic activity was assessed by MTS assay. Under both treatment conditions, SINE effectively cooperated at all concentrations with YM155 to inhibit DMPM cell growth (Figure 3A and Supplementary Figure S8). In fact, when cells were treated with the drug combinations, the inhibition of cell proliferation was consistently greater than that expected by simple additivity of the effects of the individual drugs (Figure 3A and Supplementary Figure S8). Such a synergistic interaction was drug concentration-dependent, as indicated by the progressive decrease of combination index (CI) values (Figure 3A and Supplementary Figure S8). Moreover, caspase-3 catalytic activity was consistently and significantly higher in cells treated with the selinexor/YM155 combination than in cells exposed to single agents (Figure 3B).

### Oral SINE show anti-tumor activity in DMPM xenografts

**Figure 4:**
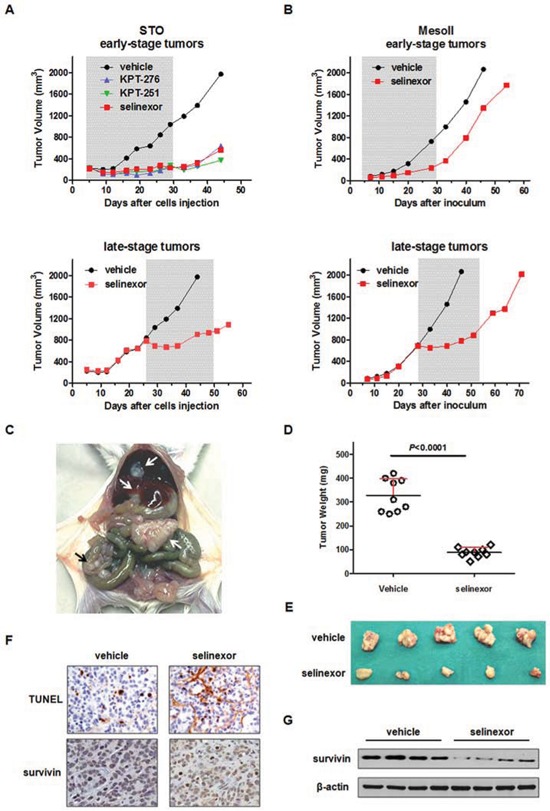
Efficacy of oral SINE against DMPM xenografts **A.** Tumor growth curves of STO cells subcutaneously injected into right flank of nude mice. Mice (eight mice/group) were randomly grouped to receive vehicle, KPT-251 (50 mg/kg, q3-4d × 8), KPT-276 (50 mg/kg, 5d/w × 3w) or selinexor (10 mg/kg, q3-4d × 8). The treatment started 4 (early-stage tumor; up) or 26 days (late-stage tumor; down) after cell injection. The treatment duration is indicated by the gray bar. **B.** Tumor growth curves of MesoII fragments subcutaneously implanted into right flank of nude mice. Mice (eight mice/group) were randomly grouped to receive vehicle or selinexor (10 mg/kg, q3-4d × 8). The treatment started 4 (early-stage tumor; up) or 28 days (late-stage tumor; down) after tumor fragments implant. The treatment duration is indicated by the gray bar. **C.** Representative photograph showing the growth pattern of STO cells following xenotransplantation in the peritoneal cavity of SCID mice. Arrow indicates the tumor mass and widespread tumor nodes. **D.** Orthotopic tumor weight distribution. Mice (nine mice per group) were randomly grouped to receive vehicle or selinexor (10 mg/kg, q3-4d × 8). The treatment started the day after cells injection and stopped 25 days after cells injection (i.e. 24 hours after the last drug treatment). **E.** Photographs of tumors from five representative mice per experimental group reported in D. **F.** Representative hematoxylin-eosin (H/E), TUNEL and survivin staining performed in FFPE sections of tumors from the experimental groups reported in D. Images for one representative mouse per group are shown. Original magnification: x40. **G.** Western immunoblotting showing the survivin expression in tumors from four representative mice per experimental group reported in D.

We next examined the *in vivo* anti-tumor activity of oral administration of SINE in DMPM xenografts. *In vivo* activity of KPT-251, KPT-276 and selinexor was initially tested against early-stage subcutaneous STO xenografts in nude mice. A remarkable and superimposable anti-tumor effect was observed after treatment with the different agents (Figure 4A and Table 2), and a stabilization of tumor volume was appreciable up to 2 weeks post drug withdrawal (Figure 4A). Although to a lesser extent compared to early-stage tumors, the clinically available compound selinexor produced a significant tumor growth inhibition even in late-stage STO tumors (Figure 4A and Table 2). In addition, selinexor significantly inhibited the growth of both early- and late-stage subcutaneous MesoII tumors (Figure 4B and Table 2). Strikingly, in late-stage STO and MesoII tumors, the growth was dramatically slowed at the beginning of the treatment, and tumor volumes kept almost constant during the course of drug administration (Figure 4B).

**Table 2:** Antitumor activity of SINE against DMPM xenografts

Cell model	Site of inoculum	Stage of tumors	Drug	Dose (mg/kg)	Schedule	TVI% (day)^a^
STO	s.c.	early	KPT-251	50	q3-4d x8	84 (33)^*^
	s.c.	early	KPT-276	50	5d/w x3w	84 (19)^*^
	s.c.	early	selinexor	10	q3-4d x8	79 (33)^*^
	s.c.	late	selinexor	10	q3-4d x8	54 (44)^*^
	orthotopic	early	selinexor	10	q3-4d x8	73 (25)^***^
MesoII	s.c.	early	selinexor	10	q3-4d x8	68 (28)^**^
	s.c.	late	selinexor	10	q3-4d x8	62 (46)^*^

Abbreviation: s.c., subcutaneous model.

a TVI% represents the maximum tumor volume inhibition % in treated *vs* control mice. In parentheses, the day on which it was assessed.

*** *P* < 0.0001,

** *P* < 0.01,

* *P* < 0.05 *vs* vehicle treated mice.

Given the strong *in vitro* synergistic activity of the selinexor/YM155 combination, we explored whether this effect was also appreciable *in vivo*. However, results obtained in early-stage STO tumors revealed that YM155 failed to appreciably improve the anti-tumor activity of selinexor (Supplementary Figure S9).

The anti-tumor activity of selinexor was further investigated in STO cells orthotopically xenotransplanted into SCID mice. Twenty-five days after cells i.p. injection (i.e., 24 hours after the last treatment), mice were euthanized and tumors were removed. At necropsy, control (vehicle-treated) mice showed a large tumor mass at the site of cell injection mainly invading the peritoneum wall, and widespread small nodules in the peritoneum and attached to the diaphragm, liver and bowel (Figure 4C), resulting in a tumor burden (average ± SD mg) of 328 ± 69 mg (Figure 4D). In selinexor-treated animals, the size of the single residual tumor mass -which was adherent to the peritoneum, in the site of cell injection- was significantly reduced (88 ± 21 mg) compared to control mice (Figure 4D, 4E and Table 2). In addition, TUNEL and survivin immunohistochemical staining of tumor sections obtained from orthotopic xenografts revealed increased apoptosis and reduced survivin expression at both nuclear and cytoplasmic cellular compartments (Figure 4F) in selinexor-treated compared to control mice. These results were further corroborated by Western blot analysis performed on frozen tumor samples (Figure 4G).

SINE were well tolerated, with no toxic deaths and minimal weight loss (<5%). In addition, no gross pathology was observed at necropsy carried out at the end of each experiment.

Taken together our findings suggest that the reduced expression of the anti-apoptotic protein survivin is a major mechanism by which SINE exert their anti-tumor activity and provide a rationale basis for offering treatment of DMPM with SINE.

## DISCUSSION

DMPM is inherently resistant to chemotherapy, which is considered a palliative treatment for patients who are not eligible for radical surgery. Currently, there is no standard systemic chemotherapy and no drug officially approved for the disease [2–4]. A limited knowledge on the dysregulated molecular pathways in DMPM, that could be specifically modulated to obtain a direct therapeutic effect or to increase the tumor sensitivity to conventional anticancer agents, also prevented the use of targeted therapeutic approaches in the clinical management of DMPM patients. These reasons underline the urgent need for new and more effective therapies for DMPM.

Based on the notion that mislocalization of proteins, which highly affect their functions, is a common feature in cancer [31], the regulation of protein trafficking between the nucleus and cytoplasm has been recently regarded as a novel control point for therapeutic interventions [32]. Clinical trials using leptomycin B, which specifically binds and block XPO1/CRM1 [33], proved to be too toxic for patients [34]. However, novel, rationally designed small molecules that form a slowly reversible covalent bond in the cargo-NES binding domain of XPO1/CRM1, i.e. SINE, (12–14) have been recently developed. In this study we demonstrated that SINE induce anti-proliferative and pro-apoptotic effects in DMPM cell lines and, most importantly, significantly inhibit the growth of subcutaneous and orthotopic DMPM xenografts at well-tolerated doses.

XPO1/CRM1 has been reported to have an increased expression in several tumor types [12, 25, 26, 35–40]. In this context, gene expression profiling analysis on clinical DMPM and normal peritoneum samples (unpublished data) showed significantly higher XPO1/CRM1 mRNA levels in tumors (Supplementary Figure S10). Interestingly, though six other members of the nuclear export protein family have been identified (XPO2-7), XPO1/CRM1 is the sole nuclear exporter for some of the major tumor suppressors (i.e., p53), cell cycle regulators (i.e., CDKN1a) and growth promoting proteins (i.e., survivin) [9–11]. *TP53* is one of the most frequently mutated genes in human cancers [41]. In this context, it has been shown that XPO1/CRM1 is able to export not only wild-type p53 but also mutant proteins which carry mutations in regions other than NES [42]. Specifically, the p53 NES lies within a highly conserved region in the C-terminal tetramerization domain (which is between the first and second of three nuclear localization signals spanning amino acids 316–325, 369–375 and 379–384 [43]), and only mutations of residues in this region prevent p53 XPO1/CRM1-mediated export [42]. In this study, we focused on two DMPM models, STO and MesoII, bearing wild-type and mutant p53, respectively. Notably, in both cell models, the p53 becomes trapped in the nucleus following SINE-mediated inhibition of XPO1/CRM1, since p53 mutations in MesoII cells do not occur in the NES.

We previously reported that survivin -as well as other members of the IAP family- is largely overexpressed in clinical DMPM [6], possibly contributing to its inherent chemoresistance, and suggested that strategies aimed at down-regulating survivin may provide a novel approach for the treatment of DMPM. Indeed, we found that siRNA-mediated survivin knockdown in DMPM cells significantly reduced their proliferative potential and enhanced both spontaneous and cisplatin- and doxorubicin-induced apoptosis. Based on this evidence highlighting a possible important function of survivin in sustaining DMPM cell growth, we explored the role of the anti-apoptotic protein as a determinant of SINE anticancer activity.

Exposure to single SINE compounds, KPT-251, KPT-276 or selinexor, induced a time- and dose-dependent inhibition of growth of the two DMPM cell lines without affecting normal cell proliferation. Such a cell growth inhibition was preceded by a decline in nuclear XPO1/CRM1 levels and an increase in nuclear accumulation of its cargo proteins p53 (in both cell lines) and CDKN1a (in STO cells only). These results were consistent with previous observations in cell lines from other human tumor types [12, 17, 20, 24–26, 28]. Here we show that survivin is an essential component in DMPM cell response to SINE-mediated XPO1/CRM1 inhibition. In fact, in both cell lines, exposure to SINE led to a time-dependent reduction of cytoplasmic survivin levels and, after an initial survivin nuclear accumulation, also to a progressive decrease in the nuclear protein abundance, through the ubiquitin-proteasomal degradation pathway, ultimately leading to the complete depletion of total survivin levels. Conversely to what was reported in TNBC cells, in which exposure to SINE repress survivin transcription by inhibiting CREB-binding mediated STAT3 acetylation and blocking STAT3 binding to the survivin promoter [22], in DMPM cell models SINE compounds failed to interfere with STAT3 acetylation status and to modulate survivin mRNA expression, suggesting that drug-induced effect on transcription could be cell-context dependent.

In both DMPM cell models, drug-induced reduction of cytoplasmic survivin levels correlated with the onset of caspase-dependent apoptosis. We further observed that SINE can be combined with other survivin inhibitors, such as the survivin suppressant YM155 -which is currently being tested in clinical trials (http://www.clinicaltrials.gov) [7]- to achieve enhanced *in vitro* growth inhibition in DMPM cells.

*In vivo* experiments with orally administered KPT-251, KPT-276 or selinexor indicated that each compound was able to significantly reduce the growth of early stage subcutaneous STO xenografts. Interestingly, additional experiments carried out with selinexor, the first-in-class SINE currently being developed for clinical use in solid and hematologic malignancies (http://clinicaltrials.gov), demonstrated that the compound was also able to inhibit the growth of late-stage subcutaneous STO and MesoII xenografts in nude mice. Most importantly, oral administration of selinexor to SCID mice reduced the growth of orthotopic STO xenografts, which properly recapitulate the dissemination pattern in the peritoneal cavity of human DMPM and, for this reason, represent a valuable model for investigating novel therapeutic approaches for the disease. Consistent with an important role of survivin as a determinant of anti-cancer activity of SINE compounds, a reduction of the protein expression was observed in tumor specimens obtained from selinexor treated mice.

Overall, our preclinical data corroborate previous evidence of an important anti-neoplastic activity of SINE compounds in experimental models of many human solid and hematologic malignancies [12, 13, 15–29] and form a solid foundation that could promote the clinical translation of SINE for the treatment of DMPM. In addition, survivin down-regulation appears as a main mechanism of SINE anti-cancer activity in DMPM experimental models, suggesting the anti-apoptotic protein as a possible biomarker for patient selection in the clinical setting. In this context, preliminary data of activity of selinexor from solid malignancy trials, together with the low toxicity profile of this class of compounds [44] and their synergistic effects in combination with other anticancer agents, further support the clinical development, also in combination regimens, of SINE against malignancies that are highly refractory to current chemotherapies, such as DMPM.

## MATERIALS AND METHODS

### Drugs

For *in vitro* studies, KPT-251, KPT-276, selinexor (provided by Karyopharm Therapeutics Inc.), and YM155 (purchased from Selleck Chemicals; #S1130) were initially dissolved in DMSO, stored at −20°C, and diluted in complete culture medium immediately before use. For *in vivo* studies, KPT-251, KPT-276 and selinexor were prepared as previously described [20]; YM155 was dissolved in sterile 0.9% saline solution.

### Cell lines

Human DMPM cell lines (STO and MesoII) were established from surgical specimens of patients who underwent surgery at Fondazione IRCCS Istituto Nazionale dei Tumori of Milan, as previously described [6]. The normal human lung fibroblast (WI38) and the normal adult human prostate (RWPE-1) cell lines were obtained from the American Type Culture Collection (ATCC; #CCL-75 and #CRL-11609). Cells were maintained in the logarithmic growth phase as a monolayer in DMEM F12 (STO and MesoII) and DMEM (WI38) media (Lonza; #12-719F and #12-604F) supplemented with 10% heat-inactivated fetal bovine serum, or in K-SFM (RWPE-1; GIBCO; #17005-042), in a humidified incubator at 37°C with a supply of 5% CO2/95% air atmosphere. Cell lines are tested fortnightly for the absence of Mycoplasma and periodically (every six months) monitored for DNA profile of short tandem repeats analysis by the AmpFISTR Identifiler PCR amplification kit (Applied Biosystems; #4322288). All cell lines were last tested in September 2014.

### Cell growth inhibition assay and drug interaction analysis

The antiproliferative activity of SINE, alone or in combination with YM155, was determined by the CellTiter 96® AQueous One Solution Cell Proliferation Assay (MTS; Promega; #G3580), as detailed in Supplementary Materials and Methods. Concentrations able to inhibit cell growth by 50% (IC_50_) and 80% (IC_80_) were determined graphically from the dose-response curves obtained after a 72-hour exposure of cells to SINE.

The nature of the interaction between YM155 and SINE was evaluated according to the method described by Chou and Talalay [45] using the CalcuSyn software (Biosoft). Specifically, combination index (CI) values <1 or >1 indicate synergy or antagonism, respectively, whereas a CI value of 1 indicates additivity. Such an interaction was also determined according to the method of Kern *et al* [46]. In brief, the expected cell survival (Sexp, defined as the product of the survival observed with selinexor alone and the survival observed with YM155 alone) and the observed cell survival (Sobs) with the selinexor/YM155 combination were used to construct an index (R)=Sexp/Sobs. R indexes >1 or <1 indicate synergism or antagonism, respectively, whereas R index of 1 indicates additivity.

### Cell cycle distribution and apoptosis analysis

Both adherent and floating cells were fixed in 70% EtOH and incubated at 4°C for 30 min in staining solution containing 50 μg/mL of propidium iodide, 50 mg/mL of RNase, and 0.05% Nonidet-P40 in PBS. Samples were analyzed with a FACSCalibur cytofluorimeter (Becton Dickinson). At least 30,000 events were read, and histograms were analyzed using the CellQuest software according to the Modfit model (Becton Dickinson).

Apoptosis was detected by using FITC Annexin V Apoptosis Detection kit I (BD Pharmigen; #556547), as detailed in Supplementary Materials and Methods. In the same cellular samples, the catalytic activity of caspase-3 was measured by means of the APOPCYTO/caspase-3 kit (MBL International; #4815). Briefly, cells were washed, pelleted, and lyzed according to the manufacturer's instructions. Total protein and the specific fluorogenic substrate N-acetyl-Asp-Glu-Val-Asp-pNA (DEVD-pNA) were mixed for 1 hour at 37°C and transferred to 96-well microtiter plates. The hydrolysis of the specific substrates was monitored by a spectrofluorometer (POLARstar OPTIMA) with 380-nm excitation and 460-nm emission filters. Results were expressed as relative fluorescence units (rfu).

### Protein extraction and western blot analysis

Nuclear and cytosolic fractions were obtained from DMPM cells using the nuclear/cytosol fractionation kit (MBL International; #JM-K266). For the assessment of the ubiquitinated form of survivin, nuclear extracts were immunoprecipitated with the anti-survivin antibody (Abcam Inc.; #ab469) for 16 hours at 4°C by addition of a 50:50 protein A slurry. Tumor protein lysates were obtained from frozen xenograft samples pulverized by the Mikro-Dismembrator II (B. Brown Biotech International).

The antibodies used in the study were CDKN1a (#ab7960), p53 (#ab26), ubiquitin (#ab7780), survivin (#ab469), β-actin (#ab8227), TBP (#ab818) (Abcam Inc.), STAT3 (#4904) and STAT3 Ac (#2523) (Cell Signaling Technology). Western blot analysis was carried out as detailed in Supplementary Material and Methods.

### ELISA assay

Survivin protein was quantified in whole or nuclear/cytosolic cell lysate obtained from cells exposed to SINE in the presence or absence of subtoxic concentrations of Bortezomib (1 nmol/L; Selleck Chemicals; #S1013) using Surveyor™ IC Human Total Survivin Immunoassay (R&D Systems; #SUV647) according to the manufacturer's protocol.

### Quantitative RT-PCR

Total RNA (0.5 μg) isolated from DMPM cells using Trizol reagent (Life Technologies; #15596-026) was reverse transcribed using the GeneAmp RNA PCR Core kit (Applied Biosystems; #N8080143) according to the manufacturer's instructions. Quantification of survivin mRNA expression levels was assessed by quantitative RT-PCR (qRT-PCR) as detailed in Supplementary Material and Methods.

### *In vivo* experiments

In both DMPM models, treatment with KPT-SINE started 4 days after tumors injection (early-stage tumors) or when tumors were ~ 800 mm^3^ (late-stage tumors). Drugs were delivered by oral gavage according to different doses and schedule determined on the basis of preliminary experiments aimed at defining the most active/less toxic conditions (data not shown): KPT-251, 50 mg/kg twice a week for 4 weeks (q3-4d/w × 8); KPT-276, 50 mg/kg 5 days a week for 3 weeks (5d/w × 3w); selinexor, 10 mg/kg q3-4d/w × 8. For combination experiments, four days after cell injection, mice (8 mice/group) were randomized to receive the drug vehicle or selinexor (p.o., 10 mg/kg, q3-4d/w × 8) and YM155 (s.c., 4 mg/kg, 5d/w × 4w), singly administered or in combination. Tumor growth was followed by biweekly measurements of tumor diameters with a Vernier calliper and tumor volume (TV) was calculated according to the formula: TV (mm^3^) = d^2^xD/2, where d and D are the shortest and the longest diameter, respectively. The anti-tumor activity was assessed as TV inhibition percentage (TVI%) in treated versus control mice, calculated as follows: TVI% = 100-(mean TV treated/mean TV control × 100).

Orthotopic model was generated by injecting 10^7^ exponentially growing STO cells suspended in 200 μl saline in the peritoneum of SCID mice. The day after cell injection, mice were randomized (9 mice/group) to receive the drug vehicle or selinexor at 10 mg/kg by oral gavage q3-4d/w × 8. Twenty-five days after cell injection (i.e. 24 hours after the last drug treatment) mice were sacrificed and the tumor masses present in the peritoneum were removed and weighted. The tumor weight inhibition percentage (TWI%) was used to assess the anti-tumor activity of selinexor. Drug treatment toxicity was determined as body weight loss and lethal toxicity. Deaths occurring in treated mice before the death of the first control mouse were ascribed to toxic effects.

At the end of each experiment, tumor specimens were fixed in 10% buffered formalin for subsequent histological and immunohistochemical analysis or flash frozen in liquid nitrogen for biochemical analysis.

The origin of subcutaneous and orthotopic xenografts was authenticated through microsatellite analysis by the AmpFISTR Identifiler PCR Amplification Kit (Applied Biosystems).

### Immunohistochemical analysis

Biogenex I6000 automated immunostainer was used to carry out immunohistochemical analyses on formalin-fixed, paraffin-embedded tumors. Antigen was retrieved by steaming with Declere™ reagent (Cell Marque; #921P). Background was blocked with Power Block™ Universal Blocking Reagent (Biogenex; #BS-1310). Primary antibody against survivin (Abcam Inc.; #ab469) was applied for 1 hour at room temperature followed by detection with the two-step, HiDef Detection™ HRP Polymer System kit (Cell Marque; #954D), followed by DAB substrate (Cell Marque; #957D). Samples were counterstained with hematoxylin, dehydrated, cleared, and coverslipped.

The TUNEL Apoptosis Detection Kit (Millipore; #17-141) was used according to manufacturers' protocol for the detection of the endonucleolytic cleavage of chromatin, characteristic of apoptosis.

### Statistical analysis

Statistical evaluation of data was done with two-tailed Student's *t* test. *P*s < 0.05 were considered statistically significant.

## SUPPLEMENTARY DATA


