# Supplementary material for: Anti-tumor activity of selective inhibitors of XPO1/CRM1-mediated nuclear export in diffuse malignant peritoneal mesothelioma: the role of survivin
Source: Oncotarget. 2015 Apr 18;6(15):13119–32. doi: 10.18632/oncotarget.3761 (PMC4537003; doi:10.18632/oncotarget.3761)
Supplement: Supplementary file 1 [file oncotarget-06-13119-s001.pdf]

# SUPPLEMENTARY DATA

## Cell growth inhibition assay

Cells were plated in 96-well flat-bottomed microtiter plates (EuroClone; #3595) for 24 hours and then treated with increasing concentrations (0.001–10 µmol/L) of KPT-251, KPT-276 and selinexor for 24, 48, and 72 hours. Control cells received vehicle alone (0.01% DMSO). For combination experiments, cells were treated with YM155 (Selleck Chemicals; #S1130) and SINE for 72 hours or 10 days. In parallel experiments, cells were pre-incubated with z-Val-Ala-Asp-fluoromethylketone (z-VAD-fmk; 50 µmol/L; MBL International; #JM-1140-1) for 6 hours before the addition of SINE. All experiments were carried out in eight replicates and repeated at least three times independently. At the end of different treatments, cell growth inhibition was determined by the CellTiter 96® AQueous One Solution Cell Proliferation Assay (MTS; Promega; #G3580), according to the manufacturer's protocols. Optical density was read at 490 nm on a microplate reader (POLARstar OPTIMA, BMG Labtech GmbH) and the results were expressed as a percentage, relative to DMSO-treated cells.

## Apoptosis analysis

For apoptosis analysis, cells were subjected to Annexin V/Propidium Iodide (PI) staining using the FITC Annexin V Apoptosis Detection kit I (BD Pharmigen; #556547), according to the manufacturer's protocol. Double staining is used to distinguish between viable (Annexin $V^-$/$PI^-$), necrotic (Annexin $V^-$/$PI^+$), early apoptotic (Annexin $V^+$/$PI^-$) or late apoptotic cells (Annexin $V^+$/$PI^+$). The resulting fluorescence was analyzed by a FACSCanto flow cytometry system (Becton Dickinson) using FlowJo Software (TreeStar Inc.).

## Western blot analysis

Cellular or tumor lysates were separated on a 4–12% NuPAGE bis-tris gel (Life Technologies; #NP0323BOX) and transferred to nitrocellulose using standard protocols. The filters were blocked in PBS 1X-Tween-20 with 5% skim milk or 5% BSA and incubated overnight with primary antibodies. The filters were then incubated with the secondary peroxidase-linked whole antibodies (Life Technologies; ECL Rabbit IgG, HRP-linked whole Ab, #NA931, and ECL Rabbit IgG, HRP-linked whole Ab, #NA934). Bound antibodies were detected using the Novex ECL, HRP Chemiluminescent substrate Reagent Kit (Life Technologies; #WP20005). Filters were autoradiographed and images were acquired by Biospectrum Imaging System (Ultra-Violet Products Ltd). β-actin and TBP monoclonal antibodies were used to confirm equal protein loading on the gel, and also to show the relative purity of the cytosolic or nuclear fractions.

## Quantitative RT-PCR

RNA purity and integrity were assessed with denaturing gel electrophoresis. The RNA was quantified spectrophotometrically and was then stored at –80°C. Reverse transcription reaction was performed using the GeneAmp RNA PCR Core kit (Applied Biosystems; #N8080143) according to the manufacturer's instructions. The PCR reaction was carried out in a volume of 10 µl containing single-stranded cDNA (2.5 µl), TaqMan Universal PCR Master Mix (5 µl; Applied Biosystems; #4304437), survivin TaqMan gene expression assay (0.5 µl; Hs00153353_m1; Applied Biosystems; #4331182) in nuclease-free water (2 µl). The reactions were performed using the ABI 7900HT instrument (Applied Biosystems). GAPDH (PN4326317E; Applied Biosystems; #402869) was used as normalizer. Amplifications were run on the 7900HT Fast Real-Time PCR System. Data were analyzed by SDS 2.2.2 software (Applied Biosystems) and reported as relative quantity (RQ) with respect to a calibrator sample using the $2^{-\Delta\Delta Ct}$ method.

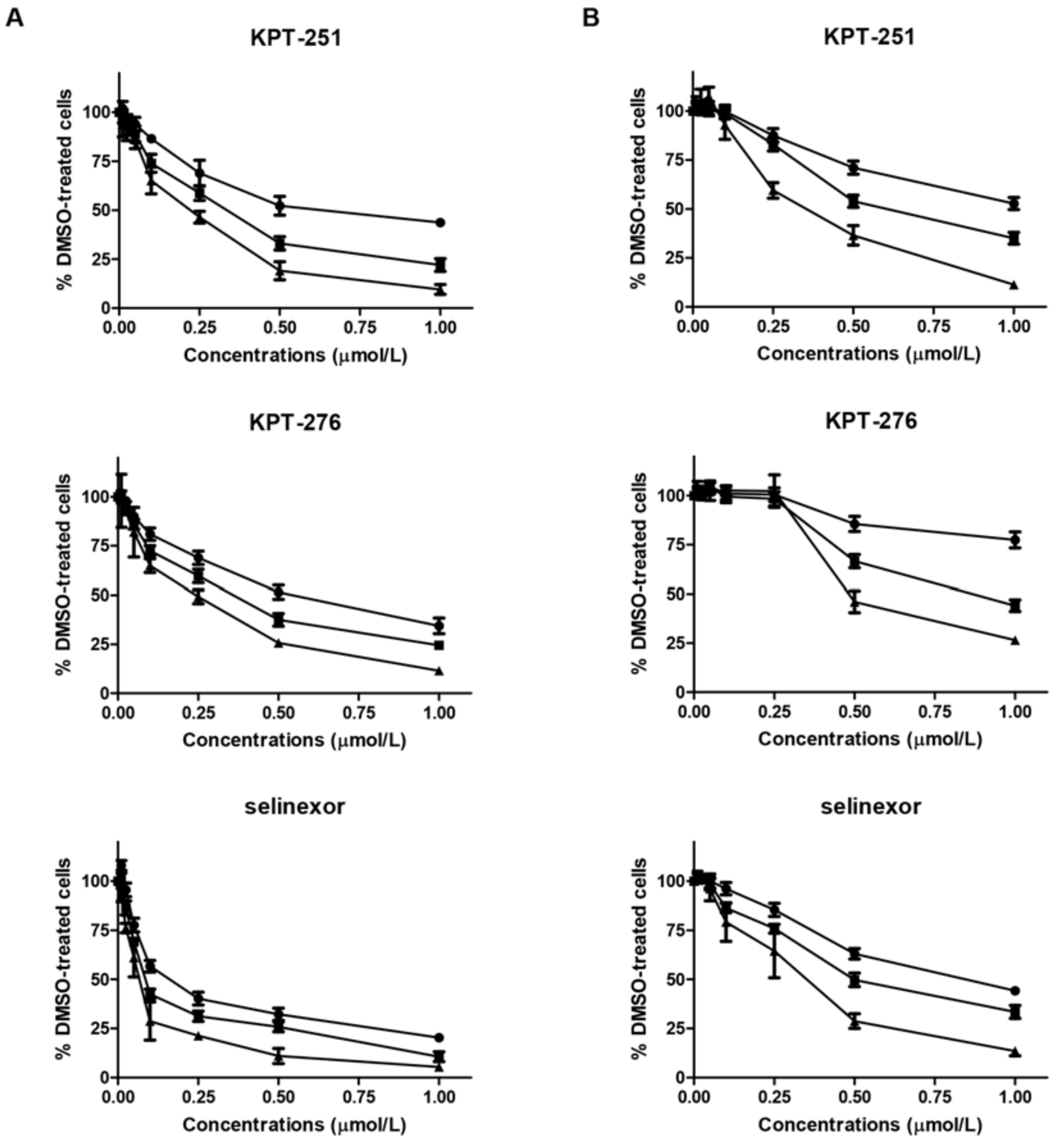


**Supplementary Figure S1: Cytotoxic activity of SINE in DMPM cell lines.** STO **A.** and MesoII **B.** cells were cultured for 24 (●), 48 (■), and 72 (▲) hours in the presence of increasing concentrations of each compound, and the cytotoxic activity was assessed by means of MTS assay. Data are expressed as mean values ±SD of at least three independent experiments.

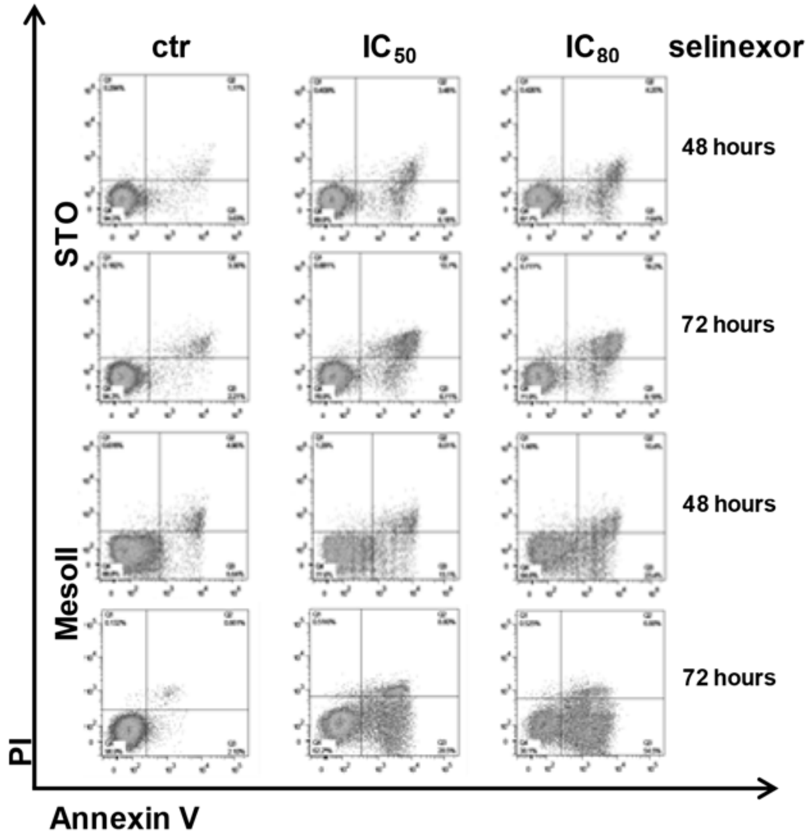


**Supplementary Figure S2: Representative dot plot analysis of Annexin V and propidium iodide (PI) staining of DMPM cells exposed for 48 and 72 hours to 0.01% DMSO (ctr) or selinexor ($IC_{50}$ and $IC_{80}$, which were determined graphically from the dose-response curves obtained after a 72-hour exposure of cells to the drug in the MTS assay).**

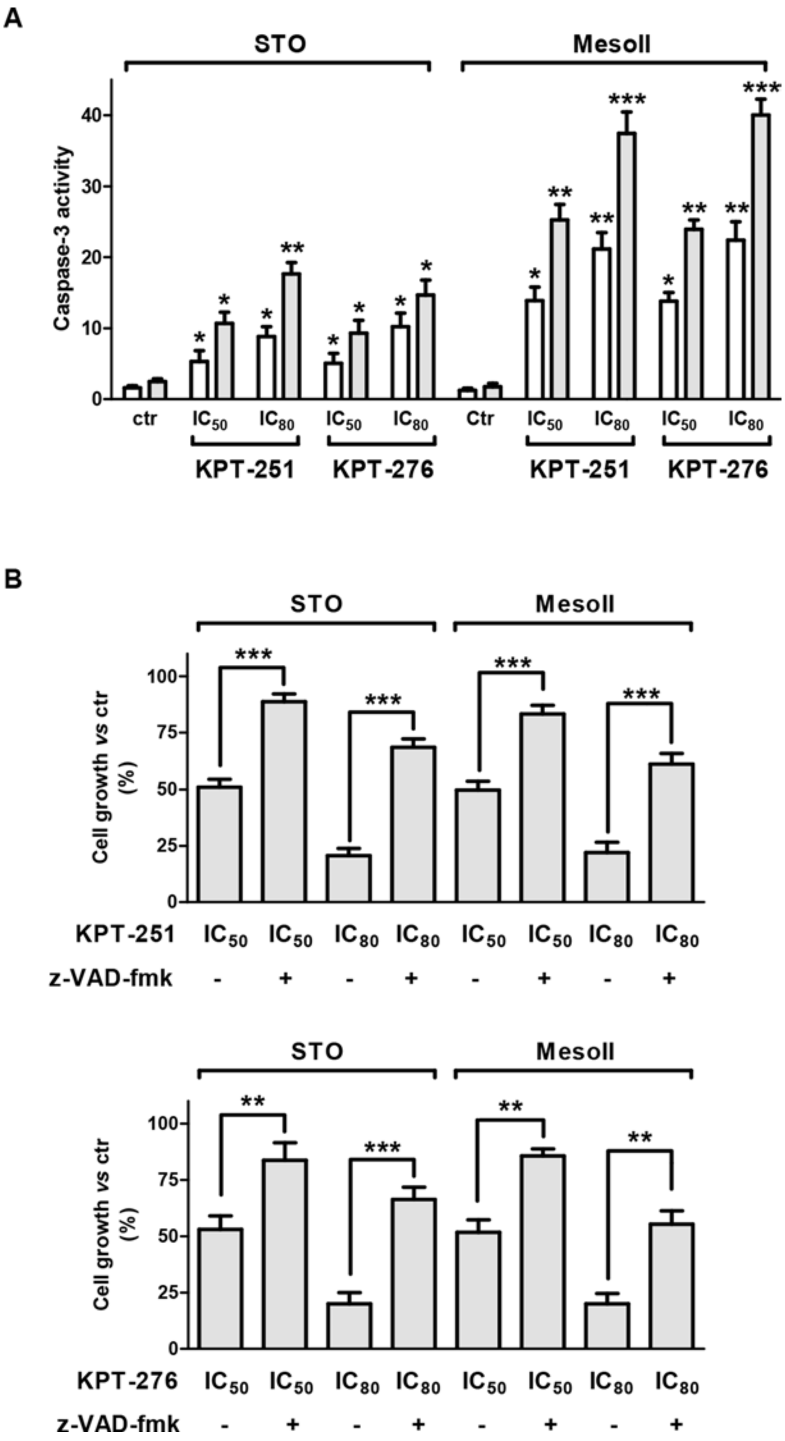


**Supplementary Figure S3: A. Assessment of caspase-3 catalytic activity 48 (black) and 72 (gray) hours after treatment with 0.01% DMSO (ctr) or KPT-251 and KPT-276 ($IC_{50}$ and $IC_{80}$, which were determined graphically from the dose-response curves obtained after a 72-hour exposure of cells to SINE in the MTS assay).** Data are expressed as relative fluorescence units and represent the mean values ±SD of at least three independent experiments. **B.** Cytotoxic effect of KPT-251 (up) and KPT-276 (down) in DMPM cells after pre-incubation with z-VAD-fmk. Data are expressed as percentage values of growth in treated cells compared with cells exposed to 0.01% DMSO (ctr), and represent mean values ±SD of at least three independent experiments ***$P < 0.001$, **$P < 0.01$, *$P < 0.05$.

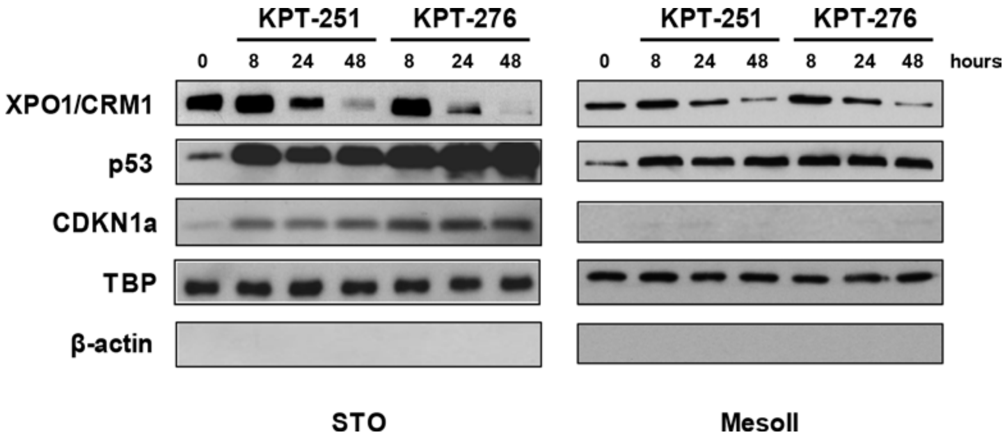


**Supplementary Figure S4: Representative western immunoblotting showing nuclear XPO1/CRM1, p53 and CDKN1a proteins in STO and MesoII cells exposed to KPT-251 and KPT-276 ($IC_{50}$, which was determined graphically from the dose-response curves obtained after a 72-hour exposure of cells to SINE in the MTS assay).** β-actin and TBP were used to confirm equal protein loading on the gel and to show the relative purity of the nuclear fractions**.**

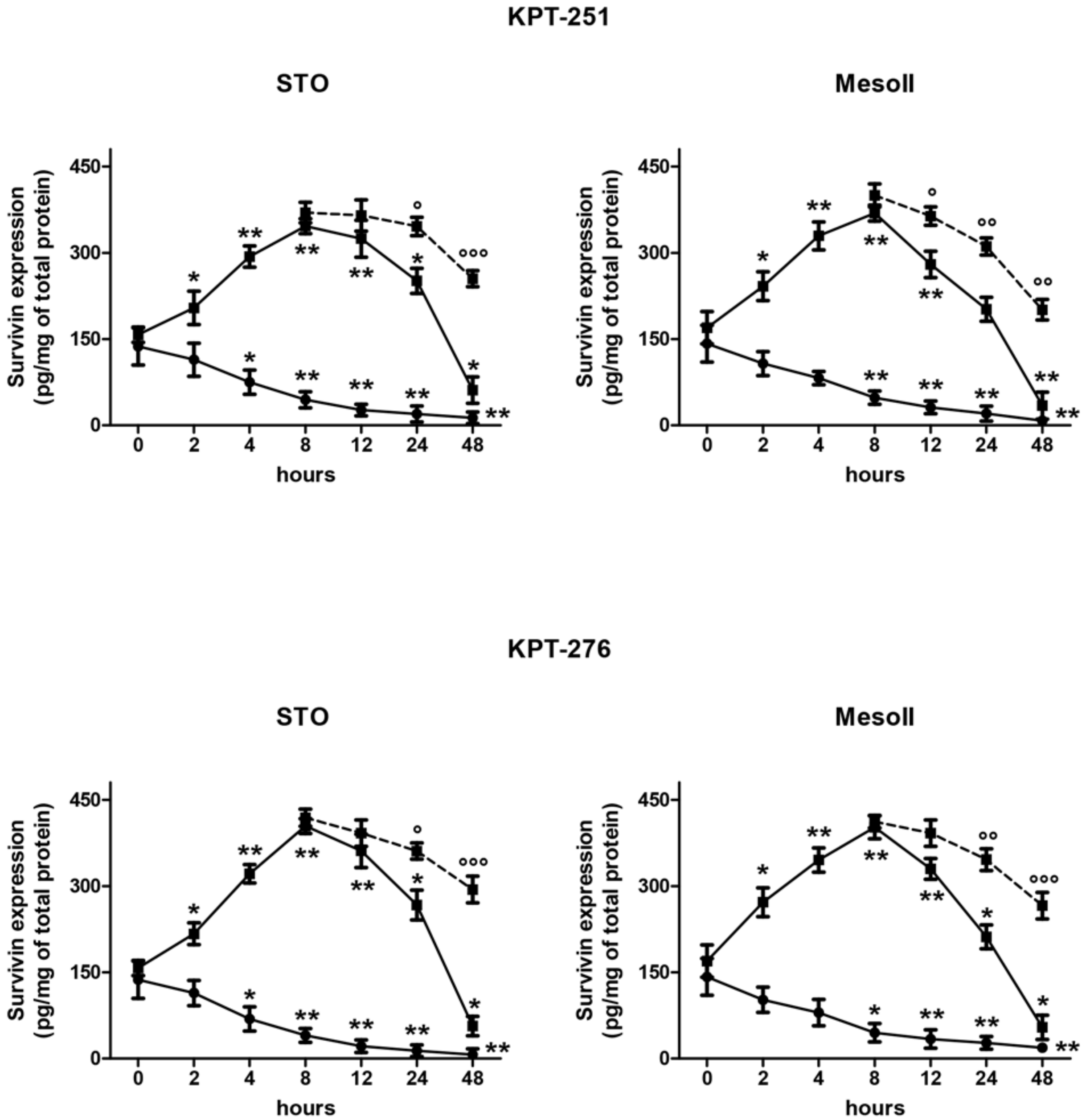


**Supplementary Figure S5: Quantification of nuclear (■) and cytosolic (●) survivin protein levels by ELISA assay in DMPM cells exposed to KPT-251 and KPT-276 ($IC_{50}$, which was determined graphically from the dose-response curves obtained after a 72-hour exposure of cells to SINE in the MTS assay) alone or in the presence of subtoxic concentrations of Bortezomib (dashed line).** Data are reported as amount (pg) of survivin normalized to total (mg) protein, and represent the mean values ±SD of at least three independent experiments. $^{**}P < 0.01$, $^{*}P < 0.05$, *vs* ctr; $^{°°°}P < 0.001$, $^{°°}P < 0.01$, $^{°}P < 0.05$, cells exposed to KPT-330 vs cells exposed to KPT-330+Bortezomib.

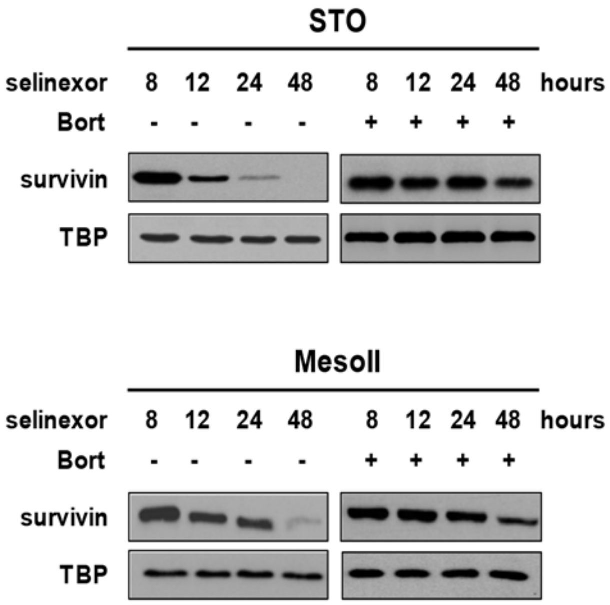


**Supplementary Figure S6: Representative western immunoblotting showing nuclear survivin protein expression in STO and MesoII cells exposed to selinexor ($IC_{50}$) ±Bortezomib (1 nmol/L).** TBP was used to confirm equal protein loading on the gel.

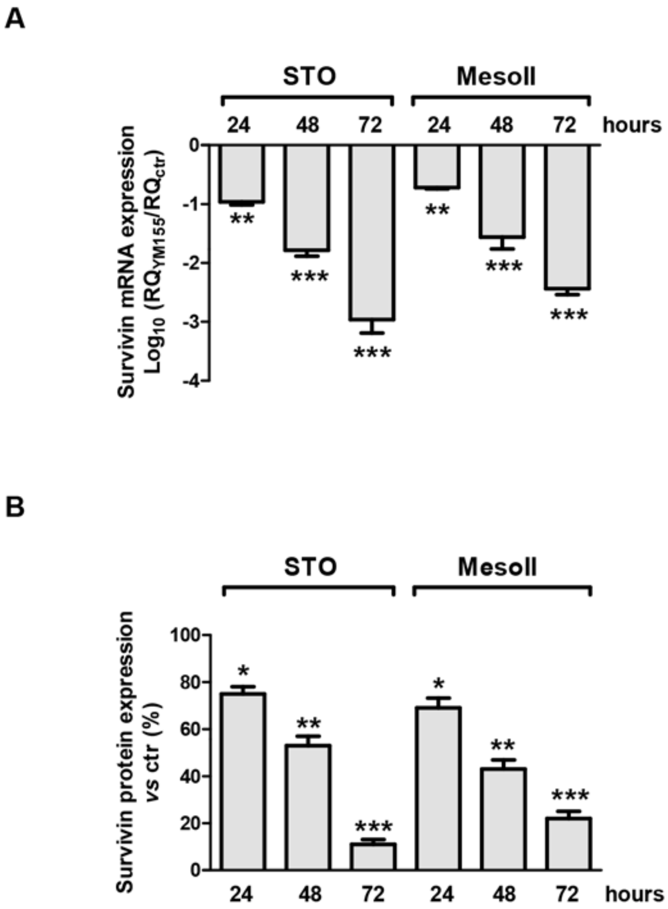


**Supplementary Figure S7: Quantification of survivin mRNA A. and protein B. expression levels (by qRT-PCR and ELISA assay, respectively) in DMPM cells exposed to YM155 ($IC_{50}$).** Data from qRT-PCR assay are reported as relative expression in YM155-treated *vs* 0.01% DMSO-exposed cells. Data from ELISA assay are reported as the percentage of survivin expression in YM155-treated cells compared with cells exposed to 0.01% DMSO (ctr). Data represent the mean values ±SD of at least three independent experiments. ****P* < 0.001, ***P* < 0.01, **P* < 0.05.

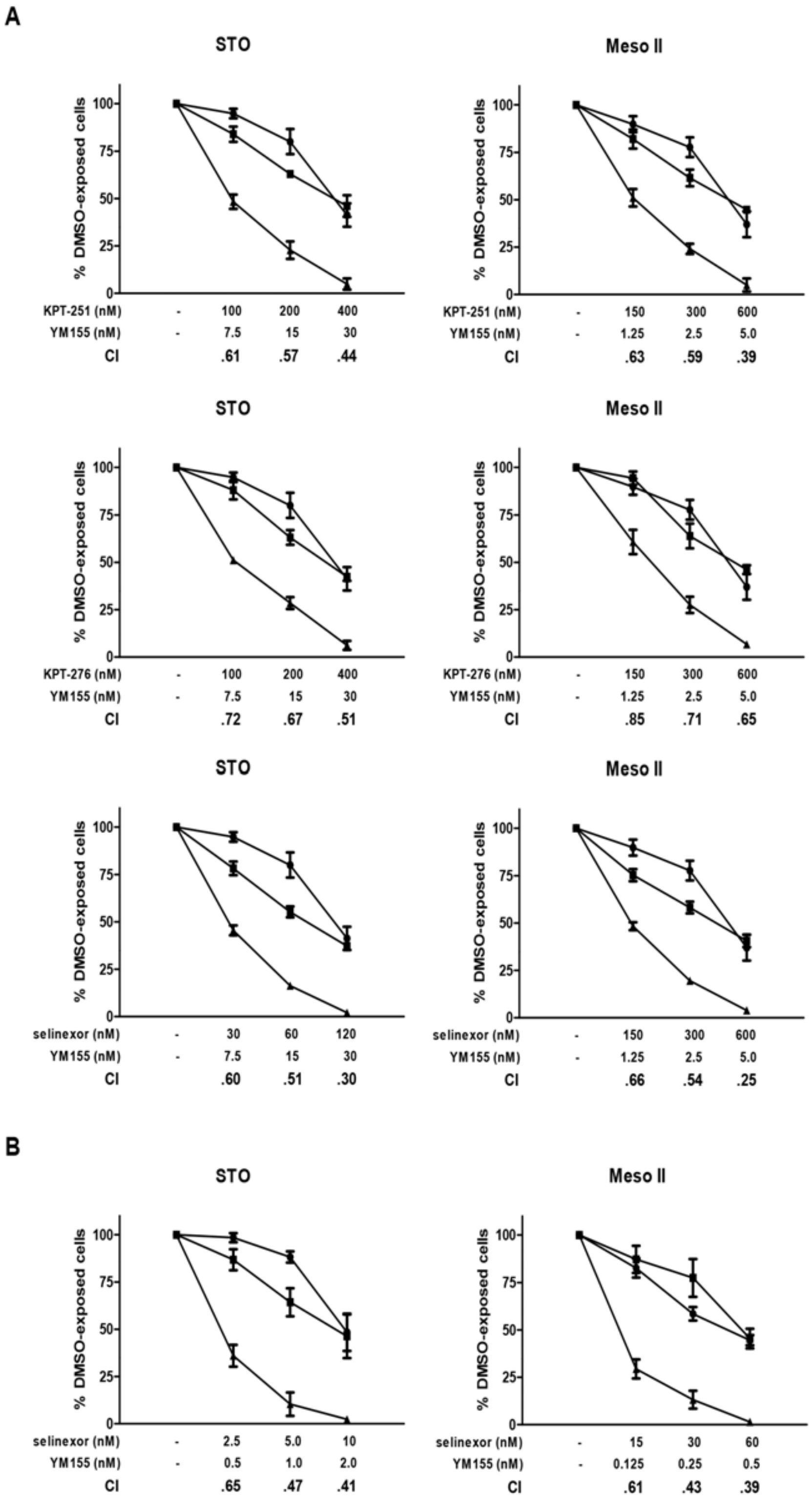


**Supplementary Figure S8: Cytotoxic effect of SINE/YM155 combination.** DMPM cells were exposed to SINE (●) and YM155 (■), alone or in combination (▲), for 72 hours **A.** or 10 days **B.** and the cytotoxic effect was assessed by MTS assay. Data are expressed as percentage values of growth in treated cells compared to cells exposed to 0.01% DMSO (ctr), and represent mean values ±SD of at least three independent experiments. CI was calculated according to Chou and Talalay [Adv Enzyme Regul. 1984; 22: 27–55].

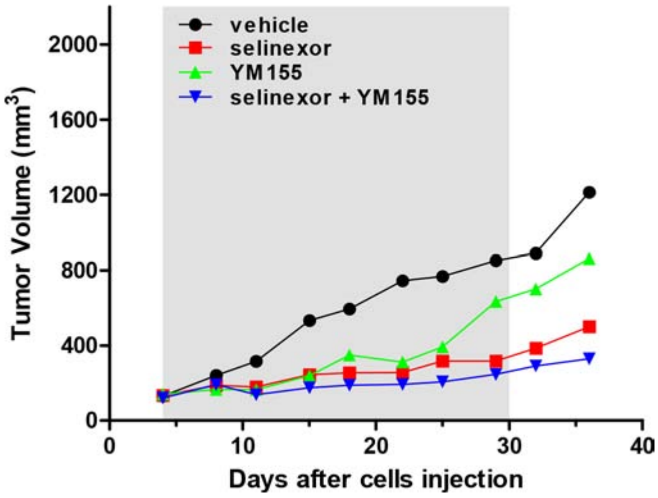


**Supplementary Figure S9: Efficacy of the KPT-330/YM155 combination against DMPM xenograft.** STO cells were subcutaneously injected into right flank of nude mice. Mice (8 mice/group) were randomly grouped to receive vehicle or selinexor (p.o., 10 mg/kg, q3-4d × 8) and YM155 (s.c., 4 mg/kg, 5d/w × 4w), singly administered or in combination. The treatments started 4 days after cell injection and their duration is indicated by the gray bar.

| UniqueID | Parametric *p*-value | FDR | Geom mean of intensities in class 'normal mesothelium' (N) | Geom mean of intensities in class DMPM 'tumor' (T) | Fold-change T/N |
|---|---|---|---|---|---|
| ***XPO1*** | 0.0044975 | 0.0398 | 864.56 | 1411.05 | 1.63 |

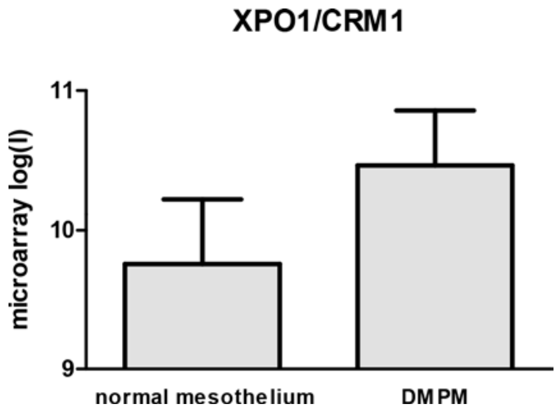


**Supplementary Figure S10: Analysis of gene expression profiles of 48 DMPM and normal mesothelium specimens on Illumina human HT12_v4 microarray platform (unpublished) revealed significant (FDR <5%) 1.63-fold overexpression of XPO1/CRM1 mRNA levels in tumor samples compared to non-neoplastic tissues, as depicted in the table (upper panel).** In the plot, XPO1/CRM1 expression data are reported as mean ± SD of log-transformed microarray intensities (I) in the two groups (lower panel).

**Supplementary Table S1.** ***TP53*** **status in DMPM cell lines.**

| Cell Lines | *TP53* Status | | |
|---|---|---|---|
| | Exon | Amino acid change | Protein effect |
| STO | // | // | wild-type |
| MesoII | 6 | P223L | mutant |
| | 8 | V274F | |
